# Association of Oral Hygiene and Periodontal Health with Third Molar Pericoronitis: A Cross-Sectional Study

**DOI:** 10.1155/2021/6664434

**Published:** 2021-02-28

**Authors:** Mehmet Gagari Caymaz, Oğuz Buhara

**Affiliations:** Department of Oral and Maxillofacial Surgery, Near East University Faculty of Dentistry, Lefkosa, Mersin 10, Turkey

## Abstract

**Background:**

Pericoronitis is a painful inflammatory condition commonly associated with third molar teeth. The purpose of this study was to investigate the relationship between oral hygiene and periodontal health status and the presence of pericoronitis in semi-impacted third molar teeth.

**Methods:**

A cross-sectional study was conducted, and 54 patients having at least one mandibular vertically semi-impacted third molar tooth with or without pericoronitis were consecutively enrolled. Subjects with pericoronitis and subjects with healthy third molars were selected according to symptoms in the gingiva overlying semi-impacted third molar teeth. Periodontal health status and oral hygiene were evaluated with the measures of plaque index (PI), gingival index (GI), and total number of sites with a probing depth (PD) ≥ 4 mm. The clinical data collected in this study was analyzed with Mann–Whitney *U* test using SPSS 20.0 package program.

**Results:**

The PI scores were found to be significantly higher in patients with pericoronitis (*p* < 0.05). Although the GI scores and PD scores were higher in patients with pericoronitis, the difference did not reach statistical significance when compared with those in healthy subjects (*p* > 0.05).

**Conclusion:**

The findings obtained in this study suggest that the amount of dental plaque was positively associated with third molar pericoronitis. Gingival and periodontal health conditions were similar between patients with and without pericoronitis. Improving oral hygiene and controlling dental plaque may help prevent third molar pericoronitis.

## 1. Introduction

Pericoronitis is a painful inflammatory condition, which is frequently observed in the mandibular third molar teeth of young adults [1]. Pericoronitis symptoms persist for several days or weeks. The condition can recur more than once within a year. The pathological condition is usually chronic [2]. As the tooth starts to erupt, the oral mucosa is perforated by the crown of the tooth, resulting in a narrow gap between the crown of the tooth and the oral mucosa that is connected to the oral cavity. This gap, also called the pericoronary gap, becomes an ideal shelter for the accumulation of bacteria and food residues. Thus, the media for the formation of pericoronary infections is formed [3]. Increased depths of periodontal pockets form an anaerobic zone leading to the emergence of periodontal pathogens. Inflammatory responses are caused by the interaction of pathogens at the biofilm gingival interface (BGI) with the individual's immune system [4].

Data regarding the frequency of periodontal diseases in patients with a semi-impacted third molar tooth are scarce, since third molar teeth are generally excluded from the analyzed data [5, 6]. There exist a considerable number of studies evaluating the relationship between asymptomatic third molars and periodontal disease. However, few studies have evaluated pericoronitis and its relationships with periodontal health. It was reported that pericoronitis may be an indicator of underlying periodontal disease [7]. Removing third molars with pericoronitis was suggested to improve the periodontal health of neighboring second molars [8].

Besides, there is no information in the literature about whether there is a difference in oral hygiene between patients with and without pericoronitis in the semi-impacted tooth. Therefore, the purpose of this cross-sectional study was to investigate the relationship between oral hygiene and periodontal health status and the presence of pericoronitis in semi-impacted third molar teeth. The hypothesis of the study was that oral hygiene and periodontal health status are associated with pericoronitis in semi-impacted third molar teeth.

## 2. Materials and Methods

### 2.1. Patient Characteristics and Inclusion-Exclusion Criteria

A cross-sectional study was carried out at the Department of Oral and Maxillofacial Surgery. The study population included 54 patients (24 females, 30 males) aged between 18 and 28 years who had at least one mandibular vertically semi-impacted third molar tooth. The patients with and without pericoronitis were consecutively enrolled among the patients admitted to our clinic. Patients with pericoronitis were selected according to symptoms in the gingiva overlying semi-impacted third molar teeth. This study was approved by the Near East University Scientific Research Ethics Committee. The current research was conducted in full accordance with the Declaration of Helsinki. All the patients were informed about the condition, and informed consents were obtained from the patients in written form.

**Figure 1:**
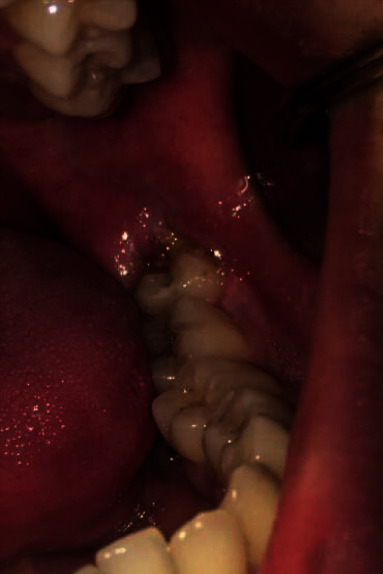
Clinical photograph showing a semi-impacted third molar with pericoronitis.

**Figure 2:**
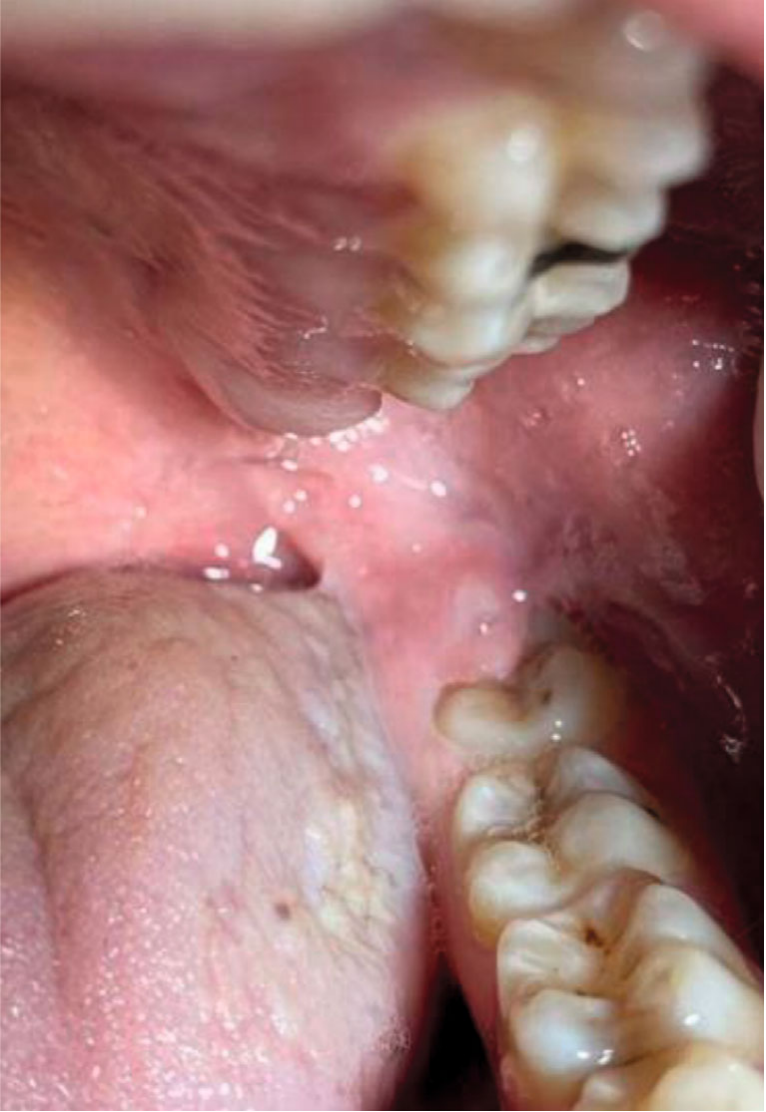
Clinical photograph showing a healthy semi-impacted third molar.

Inclusion-exclusion criteria of pericoronitis and healthy patients were as follows. In patients with pericoronitis, spontaneous pain affecting at least one vertically semi-impacted mandibular third molar, localized swelling, and mild inflammatory symptoms were sought (Figure 1). Patients with high fever (>38°), dysphagia, trismus, facial space infections, or lymphadenopathy were excluded, since these would have influenced the clinical parameters analyzed in this study. Those patients having at least 1 asymptomatic mandibular semi-impacted tooth were considered healthy (Figure 2). General inclusion criteria include ASA I and II patients (American Society of Anesthesiologists), patients not having any systemic disease, and patients having good cooperation. Patients with generalized chronic periodontal disease, a medical condition that precluded periodontal probing, patients with a body mass index more than 30, smokers, patients who underwent periodontal treatment and/or surgical procedures, pregnant patients, patients who had been using antibiotics and/or anti-inflammatory drugs within the last 6 months, and patients who were using continuous anticonvulsant, immunosuppressive, and calcium channel blockers were not included in the study. The criteria of having all other teeth in the mouth were sought except for the third molar teeth of all patients participating in the study. Routine periodontal examination of the patients was performed during the first visit before receiving any treatment. Patients with and without pericoronitis involving semi-impacted third molar teeth were examined for their oral hygiene and periodontal health status. The frequency of tooth brushing, the presence and frequency of symptoms in the past six months, and mouthwash usage of the patients were also recorded.

### 2.2. Clinical Parameters

The probing depth (PD) was measured with a periodontal probe from six different regions of the all teeth except semi-impacted third molars, namely, mesiobuccal-buccal-distobuccal-mesiolingual-lingual-distolingual regions, to determine the periodontal health status of the patients. Similarly, using the periodontal probe, plaque index (PI) scores and gingival index (GI) scores were generated from four different regions, mesial-buccal-distal and lingual/palatal, as specified in the conventional method [9, 10]. The plaque indexes for the 3rd molar operculum and the distal side of the 2nd molar tooth were evaluated separately. Evaluations on patients were performed in the same time period for each patient. All patients were examined by the same clinician. In the evaluation of the probing depth, the total number of sites presenting a probing depth of 4 mm or more was considered as the total score in the groups.

### 2.3. Statistical Analysis

The collected data were statistically analyzed with SPSS 20.0 package. Age, gender, the frequency of tooth brushing, and use of mouthwash differences between the groups were investigated with one-way analysis of variance (ANOVA) and chi-square (*χ*^2^) test, respectively. For variable comparisons between the groups, Mann–Whitney *U* test was conducted to test whether the data obtained from two unrelated samples show a significant difference. The number of sites presenting probing depths ≥ 4 mm, gingival index, and plaque index scores was analyzed in two independent groups, namely, pericoronitis and nonpericoronitis (healthy). Significance level was set at *p* < 0.05. The minimum sample size was calculated to be 52 to detect a difference of 0.50 in index scores with 80% power and an *α* value equal to 0.05 with the help of G^∗^ Power software (version 3.1.9.2, Franz Faul, Universital Kiel, Germany).

## 3. Results

A total of 54 patients with 27 pericoronitis (9 females, 18 males) with a mean age of 22.37 ± 2.04 and 27 healthy (15 females, 12 males) with a mean age of 22.22 ± 2.14 years were included in this study. No significant differences were detected in terms of age (*f*(1, 52) = 0.07, *p* = 0.795) or gender (*χ*^2^ = 2.7, *p* = 0.10) between the groups. Regular tooth brushing habit was significantly higher in the healthy third molar group (*χ*^2^ = 7.42, *p* = 0.006). There was no significant difference in the use of mouthwash (*χ*^2^ = 0.59, *p* = 0.704) (Table 1). Regarding the history of symptoms, 81.5% of the patients in the pericoronitis group stated that they had similar symptoms in the past 6 months, while only 22.2% of the patients in the healthy group had previous symptoms. To evaluate the periodontal health status in these groups, gingival index scores, plaque index scores, and the number of sites with probing depths ≥ 4 mm were analyzed. As a result of the analysis, overall plaque index scores (*p* = 0.03), plaque index scores for operculum (*p* = 0.001), and plaque index scores for distal side of the 2nd molar teeth (*p* = 0.049) were found to be significantly higher in patients with third molar pericoronitis. Therefore, the amount of dental plaque was found to be positively associated with pericoronitis. Gingival index scores and the number of sites presenting probing depths ≥ 4 mm were found to be higher in patients with pericoronitis; however, there was found to be no significant difference between the groups (*p*˃0.05) (Tables 2 and 3).

## 4. Discussion

Pericoronitis can be defined as painful inflammatory condition of soft tissues around the crown of a semi-impacted tooth. Under normal circumstances, the host defense is in balance with the bacteria. Infections may occur as a result of host defense being suppressed for any reason such as influenza, upper respiratory tract infection, and excessive fatigue. Therefore, pericoronitis may develop in semi-impacted teeth that did not cause any problems or complaints in the past [11]. Although it can be seen in all teeth, pericoronitis usually occurs around the semi-impacted mandibular third molar tooth. Wallace [12] found that pericoronitis occurs in 90% of mandibular third molar tooth that are at the same level and vertical position as the occlusal of the mandibular second molar tooth. Pericoronitis is one of the leading pathologies related to mandibular third molar teeth, and its incidence rate has been reported to be 10% [13]. Song et al. [14] reported that 72.9% of the semi-impacted mandibular third molar teeth were extracted because of pericoronitis. Periodontitis, on the other hand, is a chronic inflammatory disease that may cause destruction of the alveolar bone, cementum, periodontal ligament, and gingiva, eventually resulting in loss of teeth. As with most chronic inflammatory diseases, several risk factors are effective in the onset and progression of this disease [5, 15]. Recently, one of these factors has been reported to be erupted or semi-impacted third molar teeth [7, 16, 17].

A total of 54 patients consisting of 22 female and 32 male patients with a mean age of 21.8 years, who were admitted to the Near East University Department of Oral and Maxillofacial Surgery for treatment, were included in the study. Participants with smoking habits and systemic health conditions which might affect the periodontal status were excluded. Since the study was planned as a cross-sectional study, all the patients were evaluated at the first clinic visits. All patients were examined by the same clinician. Periodontal health status of the patients was determined according to the patient's clinical periodontal measurements [18]. The individuals with mandibular semi-impacted teeth were divided into two groups as pericoronitis and healthy, and the PI, GI, and PD measurements were obtained in order to evaluate the current status of the periodontal tissues of the patients in this group. In the existing literature, no studies evaluating the relationship between periodontal health and semi-impacted teeth with pericoronitis were encountered. Previous studies rather focused on the periodontal condition in the second and third molar regions in patients with semi-impacted third molars.

Conventional periodontal diagnostic measurements such as periodontal probing depth, gingival index, and plaque index are common measures to evaluate the severity of the disease, but these measurements are limited to show the activity of the disease. Therefore, different diagnostic methods are used to determine the activity of the disease, treatment efficacy, and course of the disease. In determining the level of periodontal disease, gingival fluid, which can be easily, reproducibly, and atraumatically obtained, is often utilized [19–21]. PI is used to measure the current hygiene habits of patients. PD measurement is performed in order to evaluate the progression of the disease or the results achieved with periodontal treatment. GI parameter is frequently used in clinical studies as an indicator of gingivitis [22]. To the best of the authors' knowledge, this study can be regarded as the first to assess the association between oral hygiene, general periodontal status, and the presence of third molar pericoronitis. Since no research could be found addressing this relationship, there were no data to compare our results with. More comprehensive studies on the subject are required.

Several different criteria were used among studies in terms of identification or selection of periodontal disease. In the study of Machado et al. [23] individuals with at least 2 periodontal pockets of PD ≥ 5 mm were considered to have chronic periodontitis. Cutler et al. [24] included individuals with 1 or more periodontal pockets of PD ≥ 6 mm into the periodontitis group and individuals with PD ≤ 4 mm into the control group. In our study, the number of sites presenting PD ≥ 4 mm was used to assess the degree of periodontal disease. This may be attributed to the different results of each study. Measurement and evaluation must be standardized in order to compare the results accurately.

Some researchers reported that third molars are as prone to periodontitis as other teeth [5, 6]. However, data from third molar teeth are often excluded from studies on periodontal pathological diseases. Later, data from Blakey et al. showed that periodontal inflammatory disease was more common in teeth adjacent to partially erupted asymptomatic third molars when compared to the teeth positioned more anteriorly [25, 26]. More than two-thirds of the subjects had at least one mandibular or maxillary third molar with PD of 4 mm or more. Large amounts of pathogens are regarded as risk factors which contribute to the progression of periodontal disease [27, 28].

The third molars are positioned at the most distal areas of each dental arch and begin to appear in the mouth at the age of 19 years after bone development is completed [29]. Anatomically, mandibular third molars are located in the alveolar bone at the junction of the corpus and ramus. Several studies reported that the number of PDs ≥ 4 mm was higher for mandibular third molars compared to the other teeth [28, 29]. Relatively late eruptions of mandibular third molars and the anatomical position of these teeth in the jaw may attribute to this high prevalence. However, in this study, no significant difference was found regarding the number of pathological probing depths between healthy and pericoronitis patients, although the number of sites presenting PD ≥ 4 mm was higher in pericoronitis patients.

Periodontal PDs obtained from 6 different sites of each tooth present an estimation of the surface area of the biofilm gingival interface and thereby indicate the potential severity of periodontal disease [4]. Deeper PDs were measured around clinically visible third molars leading to the appearance of anaerobic conditions at the biofilm gingival interface and allowing the colonization of subgingival pathogens [30, 31]. After colonization of pathogens in this region, the bacteria surrounding the third molars are hard to eradicate by using only mechanical means and the pathogens detected in the pockets around the third molars act as a potential reservoir for other pathogens to colonize around other teeth, particularly around second molars [32]. A study reported that if one PD ≥ 4 mm is identified in a third molar region, the risk of having more PDs ≥ 4 mm would increase at future follow-ups [33].

Although third molars appear to be a risk factor for early periodontal disease in some studies, there is no solid evidence to support this relationship. Even so, data obtained from 8,500 subjects suggested some conclusions. During the eruption of third molars, a greater number of periodontal diseases can be identified in the molar region of the mouth, indicating that the third molars can be a sign of unidentified periodontal inflammatory diseases. The high prevalence of at least 4 mm PD seen around the third molars and the larger mean PD seen in the first or second molar teeth when the third molars are exposed in the oral cavity have shown that the third molar teeth appearing in the mouth may be a risk indicator for the early stage of periodontal disease [17, 33]. In another study, after the extraction of third molar teeth, the clinical periodontal status of patients with mild pericoronitis was evaluated and a significant improvement was reported in the periodontal status of second molars and other teeth [8]. This may suggest that the presence of pericoronitis would affect the periodontal state in the mouth. In the present study, GI, PI, and PD values of patients with pericoronitis and healthy semi-impacted teeth were compared. As a result, a significant difference was detected in PI scores, whereas a statistically insignificant difference was found in GI scores and the number of sites with PD ≥ 4 mm. Irregular tooth brushing habit was found to be significantly higher in the pericoronitis group, which may indicate a positive effect of brushing on pericoronal health. Another finding of this study was that a higher percentage of patients in the pericoronitis group had experienced similar symptoms in the past six months compared to the healthy group. This supports the recurring nature of the pericoronitis.

When third molars become clinically visible and can be probed, the bacteria in the oral flora start to colonize on the tooth surface in a nonsheddable biofilm. Once bacteria have colonized the periodontal tissues, it is difficult to eradicate them. High numbers of pathogens are accepted as risk factors for the progression of periodontal disease [4, 30, 34]. Both Blakey et al. [35] and Rajasuo et al. [36] found high amounts of periodontal pathogens in biofilm specimens obtained from individuals with pericoronitis. In the study of Blakey et al. [35], it was found that the third molar extraction was effective in changing biofilms and the number of pathogens was reduced to the levels of control subjects. This may indicate that the presence of third molar pericoronitis increases the number of bacteria and affects the general periodontal condition of the mouth. Some studies found no association between dental plaque and pericoronitis [37, 38]. Katsarou et al. [39] reported that oral hygiene and PI scores had a weak statistical effect on pericoronitis. However, different results of plaque index were found in this study, suggesting that the amount of plaque may have an influence on pericoronitis occurrence by increasing bacterial colonization. It was also observed that the plaque scores for the operculum and distal of the second molar tooth were higher than the overall plaque scores. This finding may be related to difficulty in brushing the third molar region and brushing frequency of the patients.

Moss et al. [16] compared the mean periodontal PDs of patients with a visible third molar with those of patients with no visible third molar teeth and reported that patients with a visible third molar had a significantly deeper mean periodontal PDs. White et al. [17] stated that patients having only a visible mandibular third molar are at a higher risk of developing severe periodontal inflammatory disease than patients without mandibular third molars. In addition, Gelesko et al. [7] and Dicus-Brookes et al. [8] stated that the presence of a third molar tooth with pericoronitis forms an environment in which the bacteria reside, consequently leading to an increase in PD in third molar and second molar teeth. However, in the present study, no difference was found between patients with and without pericoronitis in terms of probing depth scores of all teeth.

## 5. Conclusions

The findings of this study suggest that the amount of dental plaque, which was evaluated with plaque index scores, was positively associated with third molar pericoronitis. Gingival and periodontal health conditions were similar between patients with and without pericoronitis. These results suggest that improving oral hygiene with effective plaque control may reduce the risk of pericoronitis in semi-impacted third molars. Patients with potential confounders such as smoking and systemic diseases were excluded which may reduce the generalizability of the results. Further studies are needed to confirm the findings obtained in this study.

## Figures and Tables

**Table 1:** Demographic characteristics of the study population.

Parameters	Pericoronitis	Healthy	*p* value
Number of patients (*n*)	27	27	—
Mean age (±SD)	22.37 ± 2.04	22.22 ± 2.14	0.795
Gender (female/male)	9/18	15/12	0.10
Regular tooth brushing (yes/no)	9/18	19/8	0.006^∗^
Use of mouthwash (yes/no)	3/24	5/22	0.704

Results are based on one-way analysis of variance (ANOVA) and chi-square (*χ*^2^) test. SD: standard deviation; ^∗^*p* < 0.05.

**Table 2:** Comparison of clinical parameters between pericoronitis and healthy subjects.

	Pericoronitis		Healthy		*p* value
	Mean	SD	Mean	SD	
Gingival index score	0.53	0.708	0.209	0.328	0.061
Plaque index score	0.846	0.737	0.453	0.424	0.030^∗^
Number of sites with probing depth ≥ 4 mm	2.63	4.877	1.333	1.544	0.372

Results are based on the Mann–Whitney *U* test. SD: standard deviation; ^∗^*p* < 0.05.

**Table 3:** Comparison of plaque index score between pericoronitis and healthy subjects.

	Pericoronitis		Healthy		*p* value
	Mean rank	Sum of rank	Mean rank	Sum of rank	
Operculum plaque index score	33.93	919	21.07	569	0.001^∗^
Distal second molar plaque index score	31.46	849.5	23.54	635.5	0.049^∗^

Results are based on the Mann–Whitney *U* test. ^∗^*p* < 0.05.

## Data Availability

The data used to support the findings of this study are available from the corresponding author upon request.
